# Supplementary material for: MetaRibo-Seq measures translation in microbiomes
Source: Nat Commun. 2020 Jun 29;11:3268. doi: 10.1038/s41467-020-17081-z (PMC7324362; doi:10.1038/s41467-020-17081-z)
Supplement: Supplementary file 10 — Supplementary Data 7 [file 41467_2020_17081_MOESM10_ESM.zip › File2/Confidence_High_Taxonomy/128235_out.krona.html]

Javascript must be enabled to view this page.

members
magnitude
magnitudeUnassigned
count
unassigned
taxon
rank

128235\_out

6

superkingdom
6
2

201174
6
phylum

class
84998
6

order
6
84999

family
84107
6

6
102106
genus

74426

SRS015217\_contig\_number\_contig-100\_3583.158350SRS019161\_contig\_number\_contig-100\_17329.167117SRS020869\_contig\_number\_33498SRS023914\_contig\_number\_contig-100\_5985.127030SRS147022\_contig\_number\_7899SRS148196\_contig\_number\_24311
6
species
